# Circulating exosomes potentiate tumor malignant properties in a mouse model of chronic sleep fragmentation

**DOI:** 10.18632/oncotarget.10578

**Published:** 2016-07-13

**Authors:** Abdelnaby Khalyfa, Isaac Almendros, Alex Gileles-Hillel, Mahzad Akbarpour, Wojciech Trzepizur, Babak Mokhlesi, Lei Huang, Jorge Andrade, Ramon Farré, David Gozal

**Affiliations:** ^1^ Section of Pediatric Sleep Medicine, Department of Pediatrics, Pritzker School of Medicine, Biological Sciences Division, The University of Chicago, Chicago, IL, USA; ^2^ Department of Medicine, Section of Pulmonary and Critical Care, Sleep Disorders Center, The University of Chicago, Chicago, IL, USA; ^3^ Center for Research Informatics, The University of Chicago, Chicago, IL, USA; ^4^ Unitat de Biofísica i Bioenginyeria, Facultat de Medicina, Universitat de Barcelona-Institut Investigacions Biomediques August Pi Sunyer-CIBER Enfermedades Respiratorias, Barcelona, Spain

**Keywords:** sleep fragmentation, obstructive sleep apnea, tumors, microenvironment, cancer biology

## Abstract

**Background:**

Chronic sleep fragmentation (SF) increases cancer aggressiveness in mice. Exosomes exhibit pleiotropic biological functions, including immune regulatory functions, antigen presentation, intracellular communication and inter-cellular transfer of RNA and proteins. We hypothesized that SF-induced alterations in biosynthesis and cargo of plasma exosomes may affect tumor cell properties.

**Results:**

SF-derived exosomes increased tumor cell proliferation (~13%), migration (~2.3-fold) and extravasation (~10%) when compared to exosomes from SC-exposed mice. Similarly, *Pre* exosomes from OSA patients significantly enhanced proliferation and migration of human adenocarcinoma cells compared to *Post*. SF-exosomal cargo revealed 3 discrete differentially expressed miRNAs, and exploration of potential mRNA targets in TC1 tumor cells uncovered 132 differentially expressed genes that encode for multiple cancer-related pathways.

**Methods:**

Plasma-derived exosomes from C57/B6 mice exposed to 6 wks of SF or sleep control (SC), and from adult human patients with obstructive sleep apnea (OSA) before (*Pre*) and after adherent treatment for 6 wks (*Post*) were co-cultured with mouse lung TC1 or human adenocarcinoma tumor cell lines, respectively. Proliferation, migration, invasion, endothelial barrier integrity and extravasation assays of tumor cells were performed. Plasma mouse exosomal miRNAs were profiled with arrays, and transcriptomic assessments of TC1 cells exposed to SF or SC exosomes were conducted to identify gene targets.

**Conclusions:**

Chronic SF induces alterations in exosomal miRNA cargo that alter the biological properties of TC1 lung tumor cells to enhance their proliferative, migratory and extravasation properties, and similar findings occur in OSA patients, in whom SF is a constitutive component of their sleep disorder. Thus, exosomes could participate, at least in part, in the adverse cancer outcomes observed in OSA.

## INTRODUCTION

Sleep is a multidimensional life sustaining function with distinct and measurable biological, behavioral, perceptual, and temporal attributes. Obstructive sleep apnea (OSA), the most common form of sleep-disordered breathing (SDB), is a highly prevalent disorder characterized by episodic changes in upper airway resistance during sleep leading to sleep disruption and intermittent hypoxia-reoxygenation, ultimately promoting increased sympathetic activity, oxidative stress, and inflammation [1, 2]. These perturbations have in turn been implicated in the pathophysiology of numerous end-organ morbidities including cancer [2]. Indeed, OSA has been associated with increased incidence of cancer [3, 4], as well as with adverse effects on cancer progression and outcomes [5]. In this setting, murine exposures to either chronic intermittent hypoxia (IH) or sleep fragmentation (SF) mimicking components of OSA lead to increased tumor proliferation and invasion [6–8], possibly via alterations in immune function and oxidative pathways [6, 9, 10].

The bidirectional communication between cancer cells and their microenvironment is critical for both normal tissue homeostasis and tumor growth [11, 12]. Interactions between the tumor and its microenvironment (e.g., endothelial cells and stromal cells, such as fibroblasts or macrophages) modulate tumor cell survival and proliferation. It has been reported that tumor exosomes are important mediators of intercellular signaling within the tumor, by transferring mRNAs, miRNAs and proteins through the fusion of multi-vesicular bodies with the plasma membrane [13, 14]. However, exosomes are also released by almost every cell in response to physiological and/or pathological signals [15], and are found in abundance in many biological fluids such as blood, saliva, and urine [16, 17], where they also mediate antigen presentation [18], immune responses [19, 20], cell migration [21], cell differentiation [22], tumor survival [23], tumor invasion [24], and angiogenesis. Thus, exosome cargo represents a potential and intriguing pathway of intercellular communication between tumor and host, particularly via delivery of miRNAs, the latter being implicated as not only modifiers of cancer cell malignant properties, but also constituting potential therapeutic targets [25–27].

Although both exosomes and miRNAs have recently become the focus of intense research, little is known about exosome cargo in the context of sleep disorders, such as OSA. We hypothesized that exosomal cargo biological effects on tumor cells will differ in SF-exposed mice, and that a restricted number of differentially expressed miRNAs in plasma-derived exosomal cargo from SF-exposed mice may be present, and potentially underlie exosome-induced alterations in cancer cell transcriptome.

## RESULTS

### SF exposures promote tumor growth and invasion in mice

In this study, mice exposed to SF revealed the presence of increased tumor growth and invasiveness as previously described [8]. Tumor volumes were significantly increased in SF-exposed mice (*n* = 12; 2,500 ± 165 mm^3^) compared to SC-exposed animals (*n* = 12; 520 ± 75 mm^3^; *p*-value < 0.0003). Similarly, tumor weight was significantly higher at day 28 in SF–exposed mice (1,448 ± 260 mg) compared to SC (514 ± 127 mg; *p*-value < 0.0001). In addition, 73% of SF-exposed animals presented evidence of invasion into the surrounding muscle compared to 0% of those exposed to SC (*p*-value < 0.001).

### Exosome cell sources

**Figure 1:**
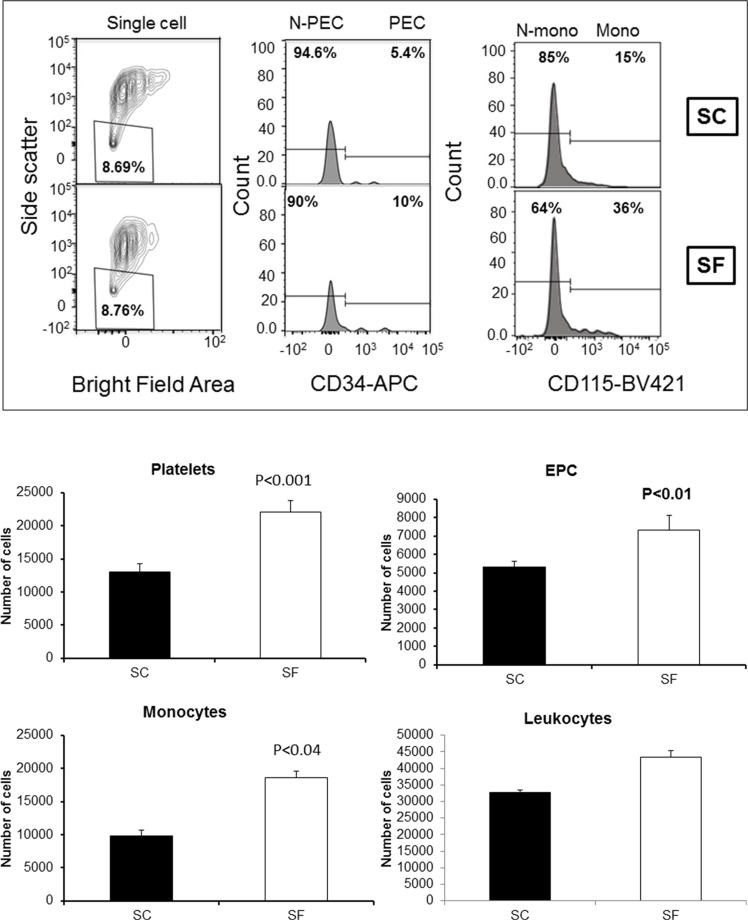
Plasma exosomal-cell sources in SF and SC using ImageStream MkII system Panel (**A**) shows a representative Imagestream analysis of exosomes derived from plasma SF(−) and SC(−). Bivariate plot of each cell was identified for platelets, leukocytes, monocytes, endothelial and endothelial progenitor cells (EPC). Platelets were identified by expression of CD41 and absence of other markers, such as CD45 and CD144. Leukocytes were identified by their expression of CD45, and that population was further subgated for expression of CD115 to identify monocytes. Endothelial cells were identified by expression of CD144, and the EPC additionally expressed CD34. ImageStream data were acquired using INSPIRE and analyzed using IDEAS. Panel (**B**) shows the quantification for exosomes from each cell source in SF and SC. (*n* = 6 for each group).

We determined cell sources from plasma-derived exosomes obtained from SC(−) and SF(−) exposed mice in whom no tumors were injected (*n* = 12/group). As shown in Figure 1, increased numbers of circulating exosomes in SF-exposed plasma emerged when compared to SC-exposed mice, and were derived from platelets (22,100 ± 1,618 vs.13,017 ± 1,194, *p*-value < 0.001), progenitor endothelial cells (7,308 ± 800 vs. 5,300 ± 433, *p*-value < 0.01), and monocytes (10,832 ± 1,234 vs. 7,782 ± 433, *p*-value < 0.04; Figure 1B). No differences in the number of exosomes originating from other leukocyte populations occurred between the groups.

### Plasma exosomes from SF-exposed mice increase tumor malignant poperties *in vitro*

**Figure 2:**
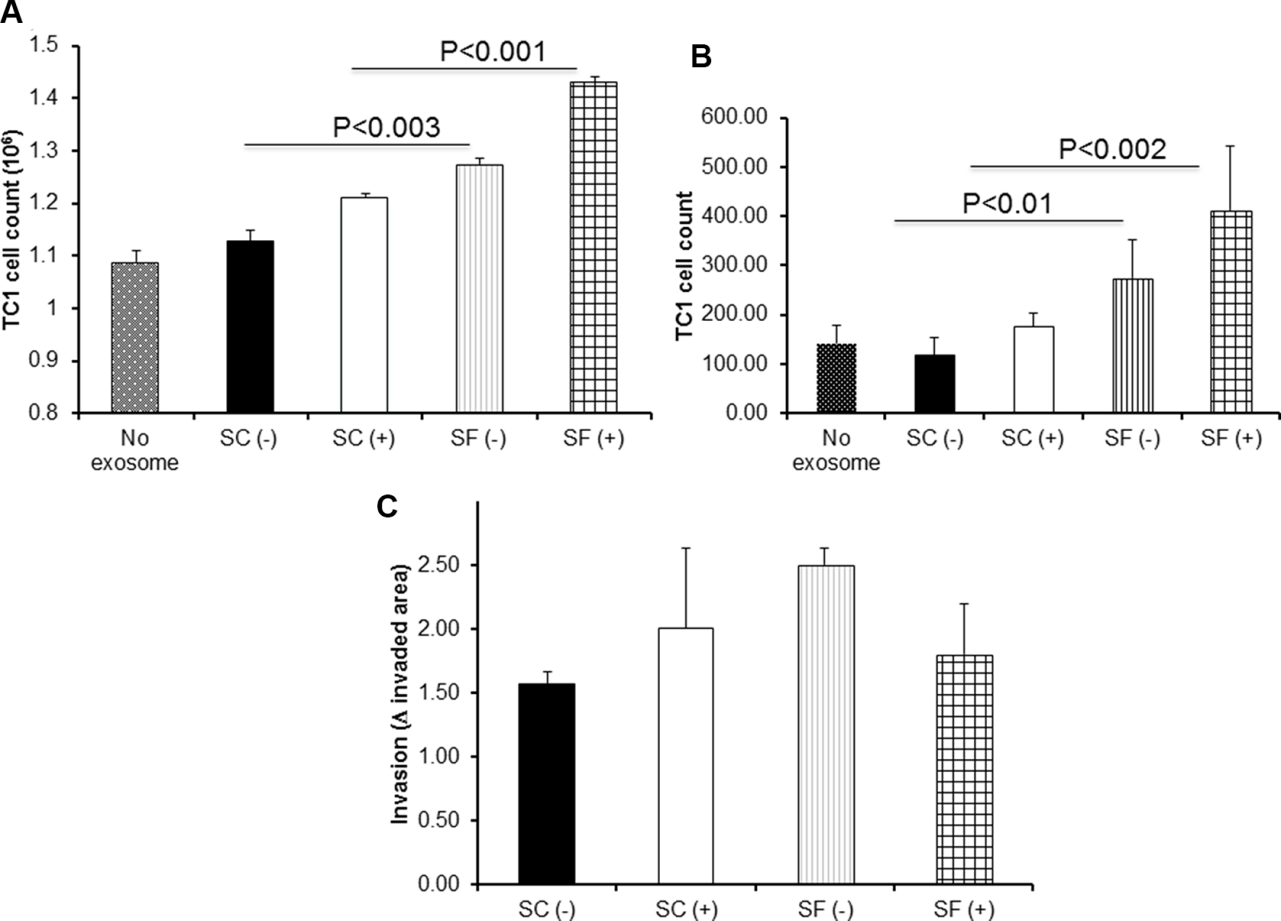
Effects of plasma exosomes derived from SF(−) or SF(+) mice on mouse lung TC1 cell on proliferation, migration, and invasion Compared to corresponding SC conditions, SF induces accelerated tumor cell proliferation and migration in TC1 tumor cells (*n* = 12) as shown in Panel (**A**) – proliferation assay, and Panel (**B**) – migration assay. The invasion of TC1 cells assessed by 3D culture panel (**C**) showed a trend towards increases invasion exerted by exosomes from SF(+) mice (*n* = 7).

We found that both exosomes from mice exposed to SF without injecting TC1 cells [SF(−)] and from SF-exposed mice injected with tumors cells [SF(+)] promoted increased TC1 proliferation *in vitro* [vs. SC(−); *p*-value < 0.001, and vs. SC(+) *p*-value < 0.003; Figure 2A; *n* = 12/group). In migration assays, co-cultures of TC1 and exosomes from SF-exposed mice increased their migration compared to those from SC-exposed mice, when fetal bovine serum was used as chemoattractant in the lower chamber of the Transwell system (Figure 2B; *n* = 12/group; SF(−) vs. SC(−): *p*-value < 0.01; SF(+) vs. SC(+): *p*-value < 0.002). However, no significant differences in the 3D-spheroid invasion assay were apparent when applying exosomes derived from SF-exposed mice with or without tumors when compared to the corresponding SC conditions (Figure 2C; *n* = 12; *p* value > 0.05).

### Plasma exosomes from OSA patients increase proliferation and migration of lung adenocarcinoma cell line

**Figure 3:**
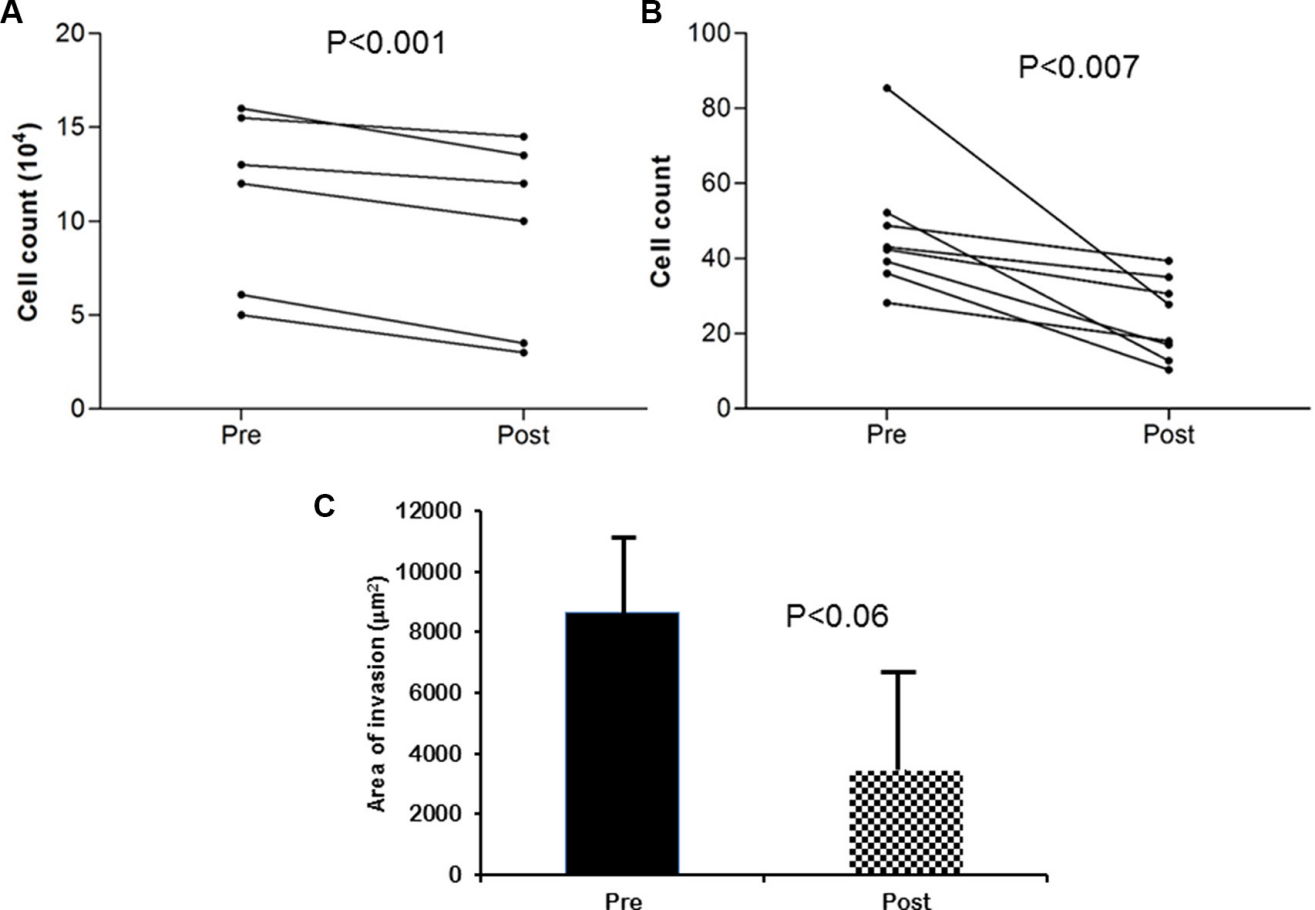
Effect of plasma exosomes from OSA patients before and after CPAP treatment on proliferation, migration, and invasion in a human lung adenocarcinoma cell line *in vitro* Exosomes-derived from patient diagnossed with OSA before (*Pre*) and after 6 wks of adherent CPAP treatment (*Post*) were incubated with human lung adenocarcinoma cell line induced increased proliferation (Panel **A**), and migration (Panel **B**), as well as statistical trend towards increased invasion (Panel **C**). (*n* = 10 patients).

Plasma exosomes derived from untreated OSA patients increased *in vitro* lung adenocarcinoma cell proliferation when compared to the same patients after 6 weeks of treatment (*n* = 10 subjects; *Pre* vs. *Post*: *p*-value < 0.001; Figure 3A) Similarly, increased migration was apparent in *Pre* compared to *Post* (*n* = 10; Figure 3B; *p*-value < 0.007). However, no differences were detected in the 3D spheroid invasion assay (*n* = 10; Figure 3C; *p*-value > 0.05).

### SF-derived plasma exosomes induce disruption of endothelial cell barrier integrity

**Figure 4:**
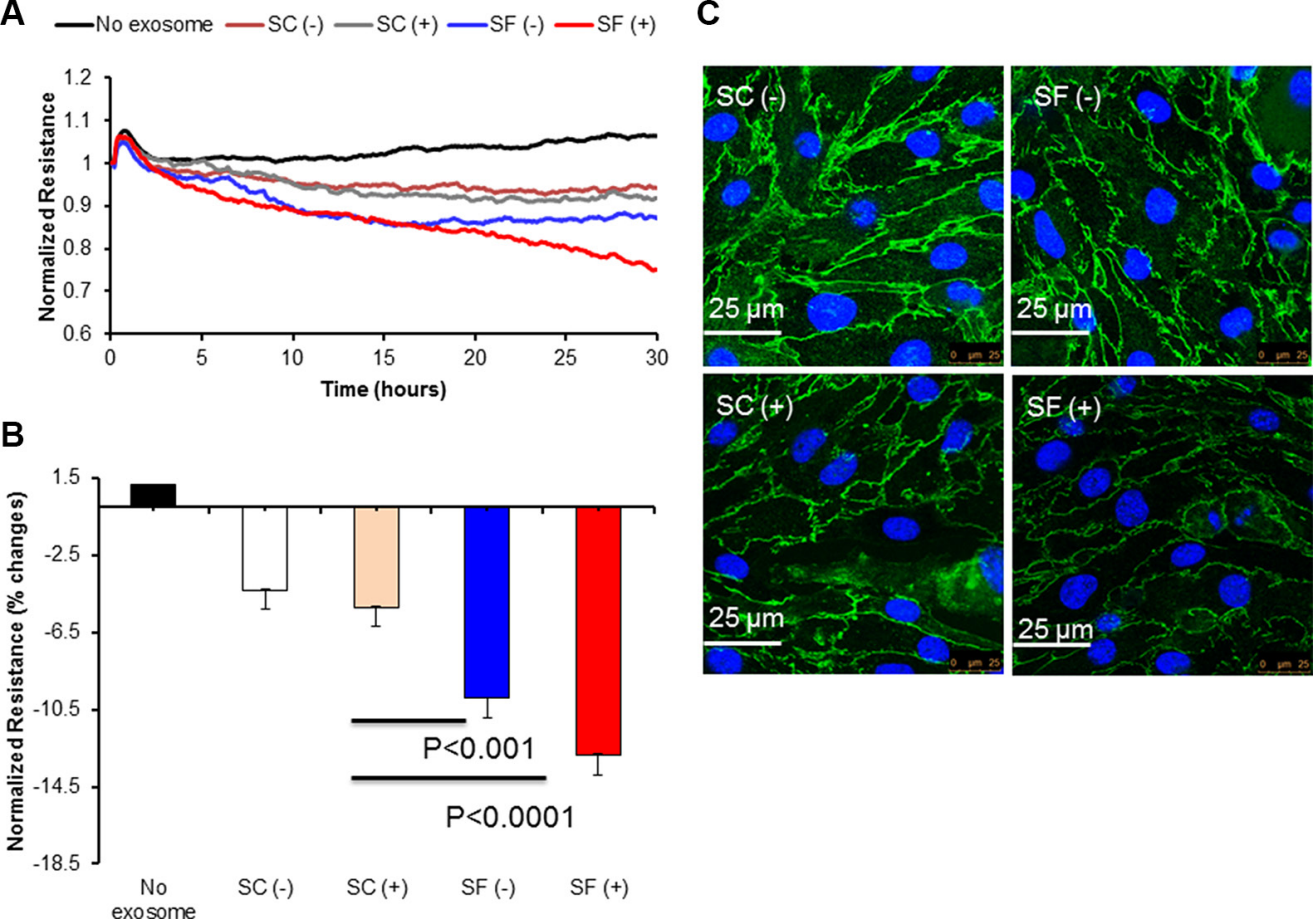
Effect of plasma-derived exosomes on Electric Cell-substrate Impedance Sensing (ECIS) and confocal microscopy imaging Time-course of normalized endothelial cells resistance (bEnd30 cells and their values were continuously monitored for up to 30 hours after adding exosomes to the confluent endothelial monolayer (Panel **A**). Plasma-derived exosomes from SF-exposed mice induce increased endothelial cell monolayer disruption compared to exosomes derived from tumor-bearing or non tumor-bearing mice exposed to SC (*n* = 8). Panel (**B**) shows values recorded at 30 hours (*p*-value < 0.001 for SF(−) vs. SC(−); *p*-value < 0.0001 for SF(+) vs. SC(+)). Panel (**C**) shows tight junction protein ZO-1 immunohistochemistry in bEnd3 cells following treatment with exosomes. Exosomes from SF and SC mice with (+) and without tumors (−) were applied to mouse bEnd3 cells for 24 hrs. Tight junction protein ZO-1 (green) and nuclei (DAPI blue) were immunostained with corresponding antibodies. Panel (C) is representative for SC(−), SF(−), SC(+), SF(+). Disruption of ZO-1 continuity is particularly apparent in SF mice with and without tumors. Images are representative of *n* = 6/group. Scale bar represents 25 μm.

Next, to test the influence of exosomes on mouse endothelial bEnd3 cells, we used two different approaches: ECIS and immunoreactivity for occludin (ZO-1) expression. Exosomes from plasma of SF-exposed mice either with or without TC1 tumors induced greater disruption of the endothelial monolayer barrier integrity compared to SC-derived plasma exosomes (Figure 4A and 4B; *p*-value < 0.001 SF(−) vs. SC(−); *p*-value < 0.0001 SF(+) vs. SC(+)). Similar qualitative findings emerged for ZO-1 immunohistochemical asessments (Figure 4C).

### Exosome miRNA cargo profiling

Using mouse miRNA arrays, we identified 26 unique miRNAs that were detectable above the background level among plasma-derived exosomes. As shown in Table 1, we found that 3 of the 26 miRNAs were significantly and differentialy expressed in SF-derived exosomes (2 miRNAs were up-regulated and 1 was down-regulated), and were further validated using qRT-PCR as shown in Table 1 (*n* = 8/experimental group).

**Table 1:** List of differentially expressed miRNAs and their validation using qRT-PCR analysis in plasma exosomes from SF(−) and SC(−)-exposed mice

miRNAs	Microarray		qRT-PCR	
	Fold change	P-value	Fold change	P-value
mmu-miR-5128	2.78 ± 0.38	0.0021	2.45 ± 0.45	0.004
mmu-miR-5112	−0.64 ±0.12	0.0039	−0.78 ± 0.14	0.001
mmu-miR-6366	1.93 ± 0.32	0.0167	1.69 ± 0.39	0.02

### Plasma-exosomal induces differentially expressed mRNA in TC1 cells

**Figure 5:**
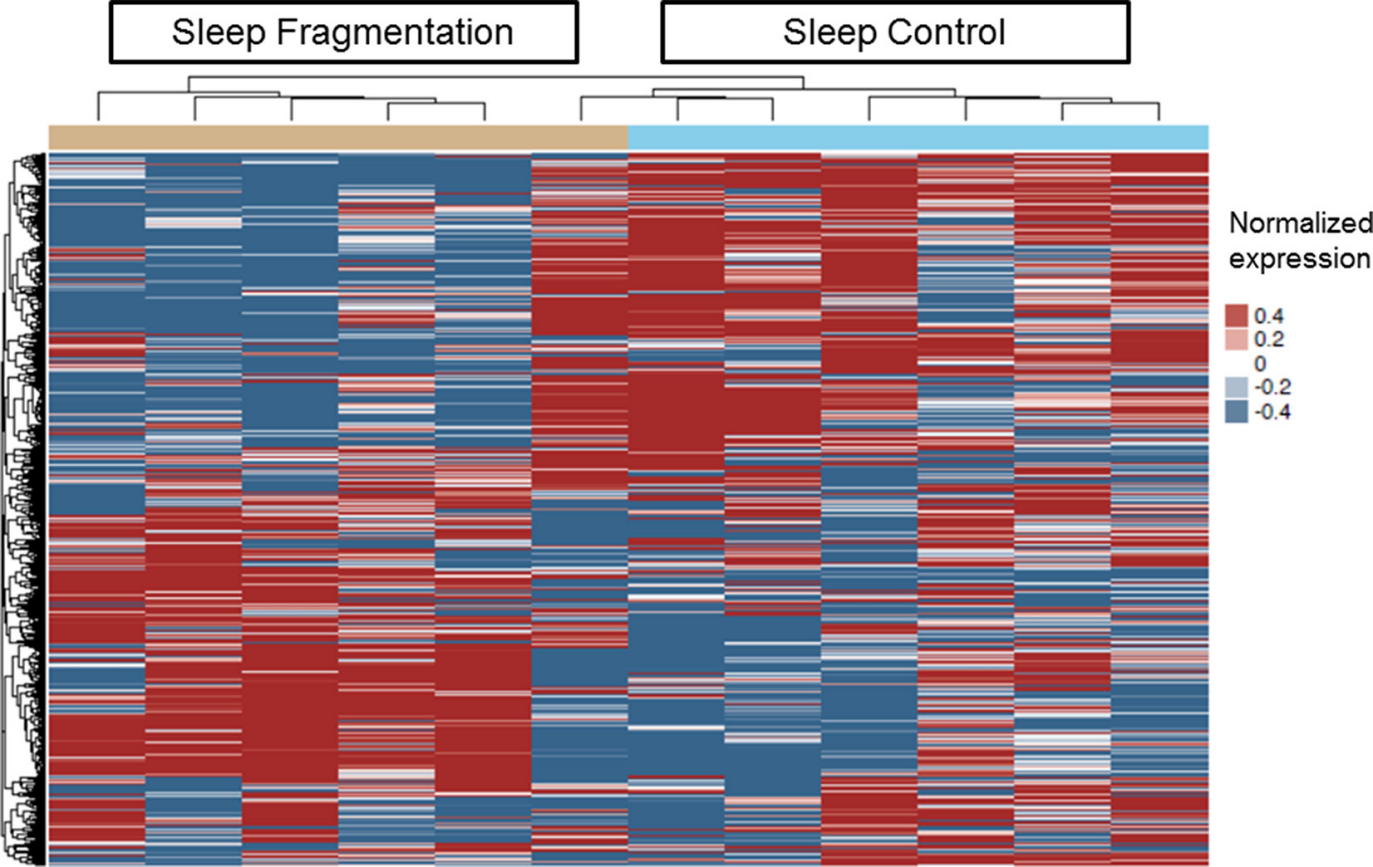
Heatmap clustering of differentially expressed genes in plasma exosomes derived from SF(−) and SC(−) treated with TC1 cells *in vitro* The color key of the heatmap represents the different expression levels. The dendrograms show one dimensional hierarchical clustering of similarities and dissimilarities in expression profiles of mRNAs to better depict distinct transcriptional patterns among SF and SC groups. (*n* = 6 per group).

Next, we used genome-wide mRNA expression analysis to identify differentially expressed genes (DEG) by exposing TC1 cells to exosomes-derived from SF- and SC-exposed mice *in vitro* (*n* = 6/group). We found 132 genes with FDR *p*-value < 0.05 as shown in Figure 5. To confirm the microarray results, qRT-PCR was performed on selected genes (*Abcf2, Ifi27l2a, Eif4g2, Mapk8ip3, Hoxb9, Rab37, Mthfr, Pdk1, Arhgef18, Mgarp, Rhot2 and Foxm1*) which were randomly selected among the differentially expressed mRNAs in the arrays, and confirmed the array findings (Table 2 and Supplementary S3). The top 50 differentially expressed genes are shown in Online Supplementary Table S1.

**Table 2:** List of differentially expressed mRNAs and their validation using qRT-PCR analysis in TC1 cells after treatment for 24 hours with plasma exosomes from SF- or SC-exposed mice

Gene Symbol	Gene name	RefSeq	Fold change			
			Microarray	p-value	qRT-PCR	p-value
Adm	Adrenomedullin	NM_009627.1	1.76 ± 0.23	1.40E-06	1.58 ± 0.11	0.002
Ankrd37	Ankyrin repeat domain 37	NM_001039562.1	−1.51 ± 0.27	4.04E-06	−0.76 ± 0.13	0.01
HLF	Hepatic leukemia factor	NM_172563.3	2.45 ± 0.36	7.05E-06	4.37 ± 0.42	0.004
Mgarp	Mitochondria localized glutamic acid rich protein	NM_026358.3	2.12 ± 0.19	0.00003	1.72 ± 0.16	0.01
Slc7a11	Solute carrier family 7	NM_011990.2	2.63 ± 0.37	0.00002	2.18 ± 0.14	0.005

Pathway enrichment analysis revealed that the top 5 canonical pathways included caveolar-mediated endocytosis signaling (*p*-value < 0.005), RhoGDI signaling (*p*-value < 0.002), heme biosynthesis from uroporphyrinogen-III (*p*-value < 0.001), semaphorin signaling in neurons (*p*-value < 0.002), and pyruvate fermentation to lactate (*p*-value < 0.003). Next, to understand the functional categories of these DEGs on TC1 cells, we first analyzed them using DAVID GO enrichment analysis and KEGG pathways. The KEGG pathways candidates found in SF compared to SC are shown in Online Supplementary Table S2.

### Integrated analysis of exosomal miRNA and mRNA expression profiles

**Figure 6:**
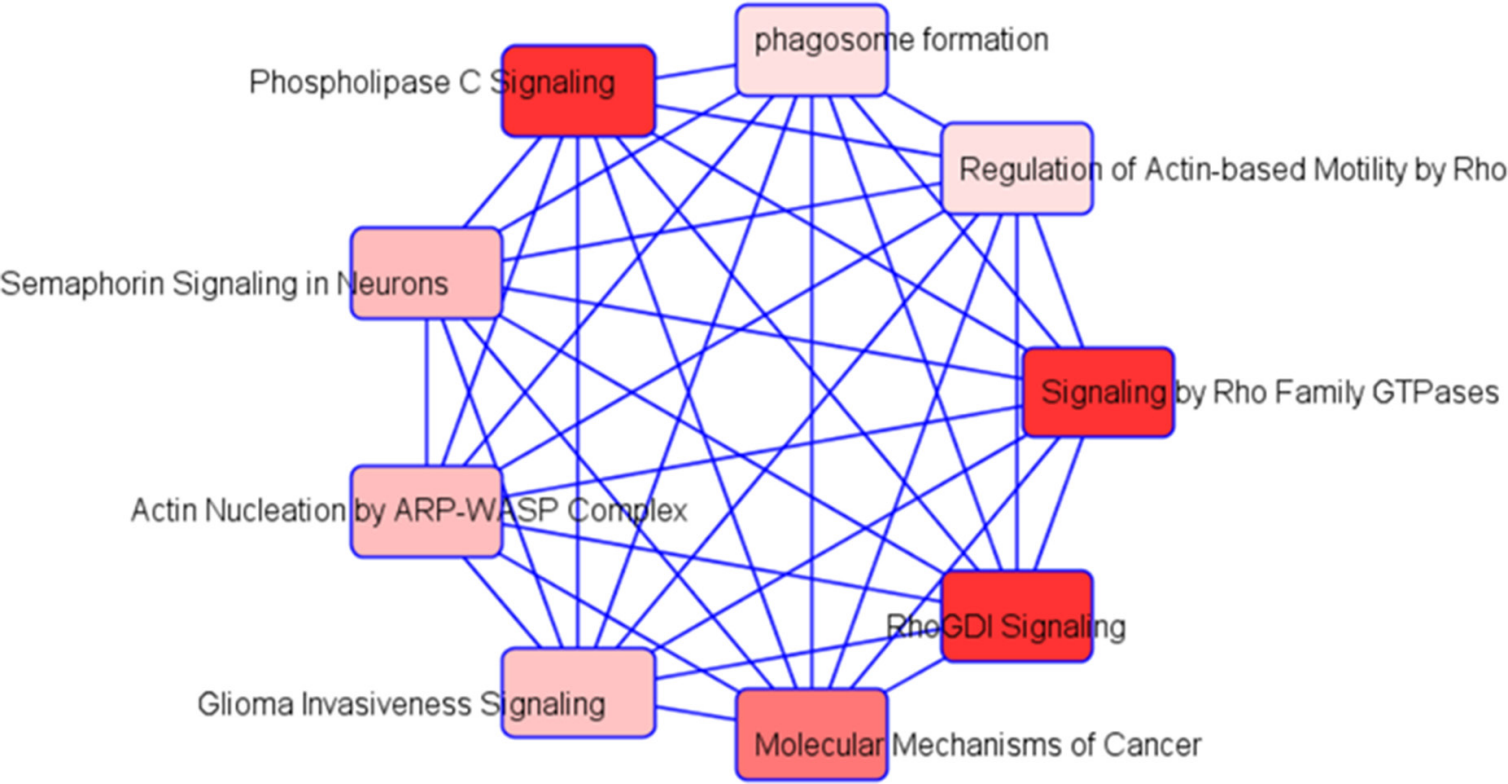
Schematic of integrative analysis of miRNA target predictions and mRNA expression in exosome-exposed TC1 cells Schema for the integrative analysis of miRNA target prediction based on miRWalk software and mRNA expression data derived from the treated exosomes derived from SF(−) and SC(−) in TC1 cells. Differentially expressed miRNA were used to identify putative gene targets and the putative mRNA targets were verified with array-based mRNA expression.

Integration of the 3 exosomal cargo miRNA putative in silico target predictions (a priori 296 theoretical genes) and mRNA expression profiling of exosome-treated TC1 cells (*n* = 132 genes) was performed and identified gene targets as follows: miR-5128 – 51 gene targets; miR-5112 – 178 gene targets; and miR-6366 – 67 gene targets, respectively; please note that there was some degree of overlap among the miRNA TC1 target genes). Supplementary Figure S1 illustrates the integration of miRNAs target predictions and mRNA gene expression from TC1 cells. To further evaluate their potential biological roles, we assessed the KEGG and GO databases using several established computational algorithms. Highly significant KEGG pathways and their genes are shown in Table 3. Five major biological processes were identified for gene ontology: GO:0044267~cellular protein metabolic process (*p*-value < 0.0004), GO:0043067~regulation of programmed cell death (*p*-value < 0.009), GO:0043069~negative regulation of programmed cell death (*p*-value < 0.01), GO:0006~protein modification process (*p*-value < 0.01), and GO:0042981~regulation of apoptosis (*p*-value < 0.01), (Supplementary Figure S2). Among the most prominent molecular function modules were: GO:00001~nucleotide binding (*p*-value < 0.00001), GO:0003824~catalytic activity (*p*-value < 0.016), and GO:0016301~kinase activity (*p*-value < 0.01), (Supplementary Figure S2). For cellular components: GO:0005737~cytoplasm, GO:0043229~intracellular organelle, and GO:0005~nuclear envelope were the most abundant (*p*-value < 0.01 for all), (Supplementary Figure S2). Using IPA software, the top networks that differentially associated with diseases and functions in SF are illustrated in Figure 6. In addition, using IPA software we identified a list of top networks identified in integrated miRNAs targets and mRNA expression including: cellular development, cell cycle, cellular assembly and organization; carbohydrate metabolism, cellular assembly and organization, cellular development; cell death and survival, tissue morphology; and cell cycle, reproductive system development and function, and cancer. The network involved in cancer is shown in Supplementary Figure S3.

**Table 3:** List of integrated KEGG pathways identified from in silico putative miRNA target genes and actual mRNA expression in TC1 cells

Category	Term	Count	%	*P*-value	Genes
mmu04622	RIG-I-like receptor signaling pathway	6	2.04	0.002	ATG5, CXCL10, MAPK12, PIN1, RELA, ATG12
mmu05215	Prostate cancer	6	2.04	0.007	PDGFRA, BCL2, RELA, LEF1, CDKN1A, IGF1
mmu04722	Neurotrophin signaling pathway	7	2.31	0.008	SHC4, PTPN11, MAPK12, BCL2, PDK1, RELA, RPS6KA6
mmu04920	Adipocytokine signaling pathway	5	1.72	0.013	PPARGC1A, PTPN11, CAMKK1, RELA, PRKAB2
mmu04110	Cell cycle	6	2.04	0.03	CDK7, SMC3, PLK1, SKP2, CDKN1A, TFDP2
mmu04510	Focal adhesion	7	2.31	0.05	PDGFRA, SHC4, SRC, ITGA10, BCL2, PPP1CB, IGF1
mmu05214	Glioma	4	1.32	0.05	PDGFRA, SHC4, CDKN1A, IGF1

We also verified the inverse correlations between the 3 SF(−) differentially expressed plasma exosomal miRNA and their actual target mRNAs in treated TC1 cells (see Supplementary Table S3). For example, mmu-miR-5112 was up-regulated in plasma exosomes from SF, resulting in reduced expression of *Arrdc3 and Fam228b.*

Next, we investigated the transcriptional regulation of miRNAs target genes, and found that different groups of target genes corresponding to transcription factors were affected in silico in SF compared to SC as shown in Supplementary Table S4. Some of these putative transcription factors are involved in apoptosis, circadian rhythm, cell differentiation, and cancer. The biological processes of some of these targets are involved in cell differentation, positive regulation of cell proliferation and inflammatory responses. However, *forkhead box M1 (FoxM1)* was the only significantly affected transcription factor identified as a putative mmu-miR-6366 target in silico that was included among the actual 132 DEGs in TC1 cells exposed to SF(−) exosomes.

## DISCUSSION

In this study, we show that plasma-derived exosomes from mice exposed to chronic SF conditions that mimic the disrupted sleep that occurs in OSA enhance the proliferative and migratory properties of TC1 lung tumor cells *in vitro*. Similar assessments using exosomes from OSA patients before and after treatment revealed essentially identical findings in a human lung adenocarcinoma cell line. We further identified miRNA exosomal cargo differences in SF that consist of 3 discrete miRNAs, whereby exploration of their potential gene targets in TC1 tumor cells uncovered ~296 genes that encode for multiple cancer-related pathways.

Emerging evidence from both animal and human studies have linked OSA with increased cancer incidence, aggressiveness, and mortality [3, 5, 7, 28, 29]. Indeed, a recent study indicated the presence of transcriptomic changes in OSA patients that pointed to cancer-related pathways [30]. To explore the potential implications of SF on tumor proliferation, we exposed mice to a SF paradigm that is non-stressful and preserves sleep duration, whereby the *in vivo* TC1 cell murine xenograft showed remarkable acceleration of the tumor proliferation and local invasiveness [8], and the present study confirms such findings, suggesting that circulating exosomes and their cargo are major contributors to SF-induced effects.

During the development and progression of cancer, the cellular composition of the tumor microenvironment is influenced by the activity of tumor cells which recruit and foster the transformation of host stromal cells into tumor supportive cells that actively participate in tumor progression [31]. Emerging evidence suggests that exosomes can interact with target cells in the tumor microenvironment by a number of mechanisms, including (1) direct stimulation of the target by surface-expressed ligands; (2) receptor transfer between the tumor cell and the target; (3) horizontal transfer of genetic information to the target, and (4) direct stimulation of the target cell by endocytic-expressed surface receptors [32, 33]. In this context, exosomes influence tumor progression, metastasis, and therapeutic efficacy, and are also responsible for stromal activation, induction of the angiogenic switch, increased vascular permeability, and immune escape [34]. As shown in our current experiments, differences in exosome functions emerged between SF and SC-exposed mice, especially influencing TC1 cell proliferation, migration, and disruption of endothelial monolayer barrier. Interestingly, plasma derived exosomes from untreated patients with OSA exhibited similar *in vitro* effects to those of exosomes from SF-exposed mice when administered to a human lung non-small cell adenocarcinoma cell line. Thus, these preliminary findings attest to significant changes in both the content and the functional properties of circulating exosomes in the context of OSA-induced disrupted sleep, further suggesting that one of the potential mechanisms underlying the adverse prognosis of OSA patients with solid tumors may reside in altered biological composition of exosomes in this disease [5, 28, 35].

Numerous studies have identified that exosomes can be released from various cell types including platelets, cardiomyocytes, endothelial cells, stem cells and tumor cell lines [36]. Here, we found that the abundance of exosomes was increased, and that the preferentially increased exosomes in plasma of SF-exposed mice originated from platelets, progenitor cells and monocytes (Figure 1). Since exosomes released by cancer cells can also signal to stromal cells within the cancer microenvironment, and impact tumor cell growth, metastasis, and angiogenesis [37–39], we examined whether the functional properties of plasma derived exosomes from SF-exposed mice differed if the mice were tumor bearing or not. Exosomes from tumor-bearing mice were always associated with increased effects compared to plasma-derived exosomes from mice without tumors, suggesting either additive or synergistic effect between SF-altered exosomes and tumor cell-derived exosomes.

Exosomes can transport and deliver a large cargo of proteins, lipids, and nucleic acids that in turn modify cell and organ function. Increasing evidence indicates that the effect of exosomes on target cells is mainly dependent on their intravesicular miRNA expression [40]. We therefore explored the miRNA content in exosomes using unbiased miRNA array approaches and identified 3 differentially-expressed miRNAs. The gene targets of these 3 miRNAs are unknown based on extensive literature searches. To identify potential gene targets, we treated TC1 cells with plasma exosomes from SF- or SC-exposed mice and using whole genome mRNA microarrays. The most highly significant genes from the bioinformatics analyses on putative target predictions of miRNAs and mRNA expression in TC1 cells are *Mthfr* for mmu-miR-5128*, Arrdc3* and *Fam228b* for mmu-miR-5112, and *Arhgef18, Foxm1, Mgarp, Pdk1,* and *Rhot2* for mmu-miR-6366. For example, genetic variations in *Mthfr* gene influence the susceptibility to occlusive vascular disease, neural tube defects, colon cancer and acute leukemia, and mutations in this gene are associated with methylenetetrahydrofolate reductase deficiency. Further exploration of the differentially expressed miRNA-predicted targets using the Sanger miRBase and using IPA software revealed that several canonical pathways that included Rho GDP-Dissociation Inhibitors (RhoGDIs). RhoGDIs are important regulators of the Rho family of small GTPases and the expression of RhoGDIs is altered in a variety of cancers in which they mediate several processes during tumorigenesis and cancer progression [41]. We also, identified 4 additional SF-modified networks, with one of these networks being involved in cancer biology (Figure 6).

In mammalian cells, miRNAs can direct the post-transcriptional regulation of target mRNAs for degradation or translational repression via binding to the 3′UTRs [42, 43], and computational predictions have shown that miRNAs may directly regulate 10–30% of protein-coding genes, on average for each miRNA can regulate the expression of several hundred genes, [44], with each miRNA repressing on average 200 transcripts [45]. Both miRNAs and transcription factors (TFs) can exert a widespread impact on gene expression. For example, genome-wide genetic analyses in many organisms have demonstrated a numerous of critical roles that TFs play in controlling gene expression during development and in homeostasis and disease [46]. In our data, we found that several transcription factors in each of the differentially expressed miRNAs. For example in mmu-miR-5128, we found *Cdip1* (cell death inducing Trp53 target 1) and this gene is involved in apoptosis and provides a new link between p53-mediated intrinsic and death receptor-mediated extrinsic apoptotic signaling, providing a novel target for cancer therapeutics aimed at maximizing the p53 apoptotic response of cancer cells to drug therapy [47]. For mmu-miR-5112, we found *Elf-1* (E74-Like Factor 1) is an Ets-related transcription factor that is expressed at high levels in T cells and is known to regulate the expression of several T cell genes, including the granulocyte-macrophage colony stimulating factor (GM-CSF) gene, the interleukin-2 receptor alpha subunit (IL-2Ralpha) gene, and the CD4 gene [48]. For mmu-miR-6366, we found that FoxM1 (forkhead box M1) was potentially involved in proliferation, invasion and migration in mouse hepatocellular carcinoma cell lines [49]. Of note, the Foxm1 gene is expressed in cardiomyocytes which is critical for proper heart development and required for cardiomyocyte proliferation and myocardial growth [50].

Several limitaitons to the present study deserve comment. First and foremost, the SF paradigm exclusively mimics only one of the constitutive elements of sleep disorders such as OSA, but does not capture the whole sleep disorder. Notwithstanding, the fact that periodic sleep disruption elicits atered exosome miRNA content and selectively enhances specific properties of tumor biology provides a strong impetus and rationale for future studies. Such studies will have to identify additional elements of exosome cargo that are affected by SF since the present study exclusively focused on miRNAs. Finally, the specific functionalities of each of the 3 miRNAs in tumor biology will have to be specifically investigated using miRNA mimics and inhibitors to uncover not only the mechanistic aspects of SF-induced modifications in cancer cell proliferation and migration, but also to develop potential therapeutic targets. In this context, our preliminary findings in patients with OSA may enable future implementation of this approach for designing precision *in vitro* reporter assays and interventions.

## MATERIALS AND METHODS

### Animals

Eight-week onld male C57BL/6 mice (*n* = 48) were purchased from The Jackson Laboratory, housed in a 12 hours light/dark cycle (light on 7:00 am to 7:00 pm) and allowed access to food and water *ad libitum*. The experimental protocols were approved by the Institutional Animal Use and Care Committee, and are in close agreement with the NIH Guide in the Care and Use of Animals. All efforts were made to minimize animal suffering and to reduce the number of animals used.

### Sleep fragmentation

The custom-designed sleep fragmentation approach used to induce disrupted sleep in rodents has been previously reported in detail [8, 51], and relies on automated intermittent mechanically-induced arousals of otherwise freely-behaving mice in a standard laboratory mouse cage, using a near silent motorized mechanical sweeper. This method does not require human contact and intervention, or the introduction of foreign objects and touching of the animals during sleep. The selected SF paradigm consisted in inducing arousals at 2-minute intervals between each sweep, implemented during the light period (7:00 a.m. to 7:00 p.m.). Four to five mice were housed in each cage to prevent isolation stress.

### Tumor cell lines and culture medium

TC1 cells (ATCC, Manassas, VA, USA) derived from primary lung epithelial cell tumors of C57/B6 mice were cultured at 37°C, 95% air, 5% CO_2_ incubator in full tumor medium as recommended by the American Type Culture Collection (ATCC). TC1 cells were cultured in RPMI-1640 medium with 2 mmol/L L-glutamine adjusted to contain 1.5 g/L sodium bicarbonate, 10 mmol/L HEPES, and 1.0 mmol/L sodium pyruvate supplemented with 2 mmol/L nonessential amino acids, penicillin and streptomycin, and 10% FBS (Life Technologies, Foster City, CA).

Mice exposed to either control sleep conditions (SC; *n* = 12) or SF (*n* = 12) were inoculated with TC1 cells (1 × 10^5^ cells in 0.2 mL PBS) by subcutaneous injection into the right lower flank. Tumors volumes were estimated every 3 days by externally measuring its length and width with an electronic caliper. After 28 days from tumor injection during which the sleep-related exposures were continued, mice were sacrificed, blood collected, and tumors excised and weighed.

The human adenocarcinoma, non-small cell lung cancer cell line was obtained from ATCC (CRL-5800, ATCC, Manassas, VA) and used for experiments with OSA patients-derived plasma exosomes (see below).

### Plasma collection and exosome isolation

Peripheral blood samples were drawn from each mouse and collected in vacutainer tubes containing EDTA (Becton Dickinson, Franklin Lakes, NJ). Blood samples were centrifuged at 2000 × g for 20 min at 4°C, and the supernatant transferred into microcentrifuge tubes and aliquoted and stored at −80°C. Exosome were isolated from frozen plasma using the Total Exosome Isolation Kit according to the manufacture's protocol (Life Technologies, Grand Island, NY). Briefly, plasma samples were centrifuged at 2000 × *g* for 20 minutes to remove cell/debris. The supernatants were precipitated by 0.2 volume of the Total Exosome Isolation Reagent (volume/volume). The mixtures were then incubated at 4°C for 45 minutes followed by centrifugation at 10,000 × *g* for 5 minutes. The supernatants were aspirated and discarded, and the exosome pellets were re-suspended in 1× PBS buffer and then stored at −80°C until used. Exosome size was determined using electron microscopy as previously described [52].

### Exosome-cell sources

Isolated exosomes from SC and SF-exposed mice were subjected to ImageStream imaging cytometry for detection of their cell sources. Briefly, 100 mL of exosomes were stained with appropriately titrated antibodies (BioLegend, San Diego, CA) as follows: CD144-PE (1:20), CD34-APC (1:20), CD41-Alexa 488 (1:20), CD115-BV421 (1:20), CD45-BV605 (1:20). First, exosomes were incubated with 5mL Fc blocking reagent (TruStain FcX - BioLegend) at room temperature (RT) for 10 minutes. An antibody cocktail of all 5 antibodies was created and queued up to 100 μl per sample by adding 75 mL per sample of Brilliant Stain Buffer (BD Biosciences, San Jose, CA). The diluted antibody cocktail was passed through a 0.1 μm filter, and 100 mL of blocked sample was added and incubated for 60 minutes at 4°C. Stained exosomes were pelleted using 17,000 × g force for 10 minutes. The pellet was suspended in exactly 100 mL of filtered wash buffer (final volume) and transferred to a sterile 96 well, U-bottom microtiter plate and loaded onto the ImageStreamX MkII (ISXMkII) image cytometer (Millipore/Amnis, Seattle, WA). The ISXMkII was set to collect fluorescence from all 5 markers as well as Brightfield (BF) and Side Scatter light (SS) using the 60X objective. Fluorescent Size Standard Kits with sizing beads (20 nm, 100 nm, 500 nm and 1000 nm) were obtained from Spherotech (Spherotech Inc. Lake Forest, IL) and used for calibration. Excitation of the various fluorophores was performed by the 4 high-powered lasers on the system, specifically, 90 mW 405 nm, 100 mW 488 nm, 200 mW 561 nm, and 120 mW 640 nm laser. 50,000 events were collected for each sample, when possible. Compensation was done using VersaComp beads (Beckman Coulter, Miami, FL) stained with the same antibodies used in the assay. Five hundred beads of each color were collected, and an auto-compensation was done using the IDEAS 6.0 data analysis software. Each gated population was interrogated via the image gallery to determine the upper and lower limits of exosomes size and shape. Using the IDEAS program, submicron sized events were gated based on BF area and Low SS intensity. Since the ISXMkII is a syringe-based, volumetric system, absolute counts (in objects/mL) are automatically calculated as part of the region statistics. These values were tabulated for each of the populations in each sample.

### Cell culture and ECIS

bEnd3 cells (ATCC, Manassas, VA, USA), an immortalized murine endothelial cell line derived from primary murine brain microvasculature transformed by polyomavirus middle T antigen was purchased from American Type Culture Collection (ATCC, Manassas, VA, USA). Cells were grown in Dulbecco's modified Eagle's medium supplemented with 4.5 g/L glucose, 3.7 g/L sodium bicarbonate, 4 mmol/L glutamine, 10% fetal bovine serum, 100 U/mL penicillin, and 100 mg/mL streptomycin, pH 7.4, and they were incubated at 37°C and 5% CO_2_ in cell culture incubator. For continuous passaging, the cells were trypsinized and centrifuged at 150 × g for 7 min, and re-plated at appropriate densities.

For Electric Cell-substrate Impedance Sensing (ECIS), bEnd3 cells were grown in DMEM media containing 2% FBS for 24 hrs. Cells were seeded (30,000 cells) and grown to confluence into ECIS arrays (8W10E, Applied Biophysics, Troy, NY) as a single confluent monolayer. ECIS monitors the impedance of small 250-μm diameter gold film electrodes used as substrates for cell growth. The next day, plasma-derived exosomes from a single mouse corresponding to each of the 4 conditions (SC-no tumor (SC(−), SC with tumor (SC(+), SF-no tumor (SF(−), and SF with tumor (SF(+)) were added to the ECIS for 30–40 hours, and the capacitance of the monolayer was monitored continuously at high frequency (40 kHz) and the resistance at a low frequency (400 Hz). Experiments for each condition were always conducted in duplicate wells.

### Immunofluorescent staining

Confluent monolayers of bEnd3 were grown on cover slips in 12-well plates for 24 hrs. with DMEM media containing 10% FBS. Following 24 hours, cells were washed with DMEM media containing 2% FBS, and maintained in the same media. Exosomes were added to cover slips for 24 hrs. Cells were fixed with 4% (w/v) paraformaldehyde in PBS for 20 min at room temperature (RT), and the cell membranes were permeabilized by incubation with 0.25% (v/v) Triton-X-100 in PBS for 10 min. After washing with PBS the samples were blocked with 3% (w/v) bovine serum albumin in PBS for 45 min at room temperature. Cells were followed by overnight incubation at 4°C with zonula occludens (ZO-1) rabbit polyclonal antibodies (1:500 Life technologies, Grand Island, NY). After a brief wash in PBS, cells were incubated with Alexa 488 anti-rabbit secondary antibodies (1:1000, 2 mg/ml; Life Technologies, Grand Island, NY) for 1 hour. Images were captured with a Leica SP5 Tandem Scanner Spectral 2-photon confocal microscope (Leica Microsystems, Inc., Buffalo Grove, IL) with a 63 × oil-immersion lens.

### Human subjects

To examine whether exosome effects induced by SF exposures cwould be replicated in humans suffering from a slepe disorder associated with substantial sleep disruption, we obtained plasma samples from 10 patients diagnosed with obstructive sleep apnea (OSA) using overnight in-laboratory polysomnography. The mean age was 52.5 ± 6.7 years, 7 were males, body mass index was 34.7 ± 4.2 kg/m^2^, and their apnea-hypopnea index, a measure of OSA severity, was 36.7 ± 8.3 events per hour of sleep (i.e., moderate to severe OSA), with arousal index being 24.8 ± 7.3/hour of sleep. All the participants provided written informed consent and the research protocol was approved by the research ethical board at the University of Chicago (protocol # 10-702-A-CR004). For each patient, blood was collected before starting continuous positive airway pressure (CPAP) treatment (*Pre*), as well as following 6 weeks of adherent CPAP treatment (*Post*). Adherence to CPAP treatment was defined as using the device at least 6 nights per week for > 5 hours per night. Polysomnographic findings during CPAP showed AHI of 2.7 ± 1.0 events per hour of sleep (*p* < 0.001 vs. *Pre*) with arousal index decreasing to 10.3 ± 3.8/hour of sleep (*p* < 0.001 vs. *Pre*). Plasma was isolated from peripheral blood samples using centrifugation at 2000 × g for 20 min at 4°C and stored at −80°C until further analysis.

### Proliferation assay

TC1 cells (2.5 × 10^4^) were cultured in 6-well plates and exosomes were added to the cells. Cultures were maintained in TC1 complete growth medium (without G418) at 37°C and 5% CO_2_ for 48 hrs. At the end, cells were harvested, labeled with CD45 and counted by flow cytometry. For human lung adenocarcinoma cell line, cells (2 × 10^4^) were grown for 72 hrs in RPMI-1640 medium modified to contain 2 mM L-glutamine, 10 mM HEPES, 1 mM sodium pyruvate, 4500 mg/L glucose, and 1500 mg/L sodium bicarbonate containing 10% FBS, for use in incubators using 5% CO_2_ in air at 37°C. Exosomes from either Pre- or Post-treatment were added to the cells every 24 hrs for 72 hrs.

### Migration test

Migration assays were performed using mouse lung TC1 cells or human lung adenocarcinoma cells in 24-well-transwell inserts with 8 μm pore size (3422, Corning, NY). TC1 cells (5 × 10^4^) were cultured on top in serum free medium either in single culture or with and without exosomes isolated from either SC or SF. Human cancer cells (2 × 10^4^) were cultured also in the top in serum free medium either in single culture, with and without exosomes isolated from *Pre* or *Post* conditions. The cells cells were allowed to migrate for 18 hours for TC1 and 40 hours for human cells from the starved medium to the lower compartment by using 10% FBS as chemoattractant. The cells were harvested after 18 hours from the bottom surface, stained with CD45 antibody, and counted by flow cytometry.

### Three-dimentional spheroid invasion test

Invasion assays were performed using TC1 cells (3 × 10^3^) or human lung adenocarcinoma cells (2 × 10^3^). Cells were seeded with exosomes and the invasion test was monitored for 6 days, using a commercially available 3D spheroid cell invasion assay (3500-096-K, Trevigen, Gaithersburg, MD). Appropriate controls included samples without invasion matrix. The 3D cultures were visualized by an Axiovert 200 M fluorescence microscope (Zeiss, Oberkochen, Germany), and phase contrast images of the spheroids were obtained at 4×magnification. SlideBook 5.5 software (Intelligent Imaging Innovations, Denver, CO, USA) was used to measure the radius of the invasive structures to determine the extent of 3D by a blinded investigator. The area of invasion was determined as the area of the spheroid embedded in the invasion matrix minus its corresponding control without invasion matrix.

### Total RNA and miRNAs isolations

Total RNA was isolated from TC1 cells treated with exosomes isolated from mice exposed to SC(−) (*n* = 6) or SF(−) (*n* = 6) using miRNeasy Mini kit (Qiagen, Turnberry Lane, Valencia, CA). Total RNA containing miRNAs were isolated from plasma exosomes SC(−) and SF(−) using the miRNeasy Serum/Plasma Kit according to the manufacturer's instructions (Qiagen, Turnberry Lane, Valencia, CA). The RNA quality and integrity were determined using the Eukaryote Total RNA Nano 6000 LabChip assay (Agilent Technologies) on the Agilent 2100 Bioanalyzer. The quality of miRNA was determined using Agilent Small RNA Kit according to the manufacturer's protocol. Both total RNA and miRNA samples were quantified on a Nanodrop 2000 (Ambion, Austin, TX).

### Exosomal miRNAs microarrays profiling

Total RNA containing miRNAs (100 ηg) from a total of 16 plasma exosome samples (SC(−): *n* = 8 and SF(−): *n* = 8) were reverse transcribed. Each sample was prepared according to the Agilent's miRNA recommended approach using the one-color technique. miRNA was dephosphorylated with calf intestine alkaline phosphatase (GE Healthcare Europe GmbH), denatured with dimethyl sulfoxide, and labeled with pCp-Cy3 using T4 RNA ligase (GE Healthcare Europe GmbH). The labeled miRNAs were hybridized to custom 8 × 60 K mouse microarrays, consisting of 60-mer DNA probes synthesized *in situ* that represent 2006 miRNA (Version 19.0) and each of them represented by 40 features probes (Agilent), Santa Clara, CA). After hybridization and washing, the arrays were scanned with an Agilent microarray scanner using high dynamic range settings as specified by the manufacturer. Microarray results were extracted using Agilent Feature Extraction software (v12.0). Bioconductor package AgiMicroRna [53] was used to perform the data preprocessing and limma [54] was also used for the differential expression analysis. The *p*-values were corrected for multiple testing by false discovery rate (FDR) using Benjamini-Hochberg procedure [55].

### Target gene predictions and analysis

Gene targets for differentially expressed miRNAs were initially computationally examined for miRNA target-prediction programs using miRwalk (http://zmf.umm.uni-heidelberg.de/apps/zmf/mirwalk2/) with 12 predicted target softwares (miRWalk, Microt4, miRanda, mirbridge, miRDB, MiRMap, miRNAMap, Pictar2, PITA, RNA22, RNAhybrid, and Targetscc). In order to improve the reliability of the miRNA targets, only target genes predicted by at least 5 of the programs were selected. The Gene Ontology (GO) terms and Kyoto Encyclopedia of Genes and Genomes (KEGG) pathways of target genes were analyzed using the online DAVID program (http://david.abcc.ncifcrf.gov/). The web-based computational tool DIANA-mirPath v2.1 [56] was used to predict the target genes and altered pathways among differentially expressed miRNAs.

### mRNA microarrays for assessment of exosome targets in TC1 cells

Total RNAs were isolated from TC1 cells treated with exosomes from SC(−) (*n* = 6) and SF (−) (*n* = 6). Briefly, purified total RNAs were processed for labeling using the Low RNA Input Fluorescent Linear Amplification Kit (Agilent Technologies, Santa Clara, CA) and hybridized with whole-genome mouse Agilent microarrays (8 × 60 K). Equal quantities of total RNA (25 ηg) for each sample were labeled, and the quality of each cRNA sample was evaluated using 2100 Bioanalyzer (Agilent Technologies, Santa Clara, CA). Each sample was hybridized to an Agilent oligonucleotide microarray for all of the 12 independent experiments. The microarray slides were scanned using Agilent dual-laser Microarray Scanner and the digitized images were acquired and processed using Agilent Feature Extraction (FE) software v.12.0. The background corrected data were normalized between arrays using cyclic Loess method [57].

We applied limma moderated *t*-test to detect differentially expressed genes, considering the batch effect caused by different chips as covariates in the linear model. *P*-values were adjusted by Benjamini-Hochberg method [55]. The differentially expressed data were analyzed using Ingenuity Pathway Analysis (IPA) software (Ingenuity Systems, Redwood City, CA) to perform functional classifications comparing SC and SF conditions. Pathway analysis and Gene Ontology (GO) analysis were performed to reveal the biological functions of differentially expressed genes.

### Integrated analysis of miRNA and mRNA expression profiles

Integrated analysis of the miRNA putative targets and mRNA expression profiles derived from TC1 treatments were carried out using the differentially expressed miRNAs and mRNAs. The predicted mRNAs were initially computationally predicted by miRNA target-prediction programs using miRwalk (http://zmf.umm.uni-heidelberg.de/apps/zmf/mirwalk2/) as described above. We used the negative regulation relationship between the miRNAs and mRNAs to associate the differentially expressed miRNAs and mRNA expression data, using FDR, *p*< 0.05 and fold-change 1.5. The Agilent identifiers of the FDR significant probes were uploaded and mapped to genes in the Database for Annotation, Visualization and Integrated Discovery (DAVID) for functional annotation. Also, the data were uploaded into Ingenuity software (Qiagen). For the transcription factors in the miRNA-mRNA interaction network, we used AnimalTFDB (www.bioguo.org/AnimalTFDB/download_index.php?spe=Mus%20musculus). These transcription factors were annotated in the miRNA-mRNA interaction networks using mygene (http://mygene.info; [58]).

### Validation of miRNA and mRNA expression

Quantitative real-time polymerase chain reactions (qRT-PCR) were utilized for assessment of mRNA and miRNA expression using the ABI PRISM 7500 System (Applied Biosystems, Foster City, CA). Total RNAs were validated using SYBR Green Master Mixes (life technology) according to the manufacturer's instructions. Primers for gene of interest were designed using Primer 3 software (http://bioinfo.ut.ee/primer3-0.4.0/), and each primer made was synthesized by Integrated DNA Technologies (IDT, Coralville, IA). Total RNA of 500 ηg was reverse transcripted into cDNA using a High-Capacity cDNA Archive Kit (Applied Biosystems, Foster City, CA). Expression levels of glyceraldehyde-3-phosphate dehydrogenase (GAPDH) were used for normalization levels. All experiments were performed in triplicates. The cycle number (Ct) values were averaged and the differences between the housekeeping gene Ct and the gene of interest Ct were calculated to measure the relative expression of the gene of interest using the 2^−ΔCT^ method [59]. The results are presented as fold change (SF relative to SC conditions).

For miRNAs, selected miRNAs were reverse transcribed with looped miRNA-specific reverse transcriptase (RT) primers (Applied Biosystems, Foster City, CA) using the TaqMan microRNA mouse assay according to the manufacturer's protocol. Briefly, RT reactions were performed in a volume of 15 μl, and each reaction contained 10 ηg of enrich miRNA. RT reactions were performed on a 7500 (Applied Biosystems, Foster City, CA). Reactions without addition of reverse transcriptase were performed alongside cDNA synthesis of each sample and used in subsequent procedures to control for potential genomic DNA contamination. All TaqMan assays were run in triplicate using TaqMan Universal PCR Master Mix II without UNG (Applied Biosystems, Foster City, CA). The qPCR results were normalized against an internal control (RNU6), and then expressed as fold changes.

### Statistical methods

For tumor malignancy related variables measured *in vitro*, 2-Way ANOVA were conducted with treatment (SC vs. SF) followed by post-hoc tests. Differences were determined by Student *t* tests or ANOVA with 2-tailed *p* < 0.05 accepted as statistically significant. Data are presented as mean ± SD. Statistical analyses were conducted using SPSS 21.0 software for Windows (IBM, Armonk, NY).

## CONCLUSIONS

The present study confirmed our previous findings that SF increases tumor proliferation, migration and invasion *in vivo*. More importantly, we found that circulating plasma exosomes obtained from either sleep fragmented mice or OSA patients enhance tumor cell line proliferation and migration *in vitro* indicating that these exosomes may serve as vehicles of cell–cell communication that underlie adverse cancer prognosis in the context of perturbed sleep. We also identified a unique set of exosomal miRNAs in SF-exposed mice that further raise the possibility that tumor cells and surrounding stroma may be altered via transfer of exosomal miRNAs. There is no doubt that improved understanding of the complex network of genes and cellular signaling transduction pathways regulated by exosomal miRNAs in the context of SF will augment our knowledge on the potential effects of OSA in cancer patients.

## SUPPLEMENTARY MATERIALS





## Abbreviations

SF: Sleep fragmentation
SC: sleep control
OSA: obstructive sleep apnea
FBS: fetal bovine serum
PBS: phosphate-buffered saline
ECIS: Electric Cell-substrate Impedance Sensing
DMEM: Dulbecco's Modified Eagle Medium
KEGG: Kyoto Encyclopedia of Genes and Genomes
GO: Gene ontology
DEG: differentially expressed genes
