# Supplementary material for: Circulating exosomes potentiate tumor malignant properties in a mouse model of chronic sleep fragmentation
Source: Oncotarget. 2016 Jul 13;7(34):54676–90. doi: 10.18632/oncotarget.10578 (PMC5342372; doi:10.18632/oncotarget.10578)
Supplement: Supplementary file 1 [file oncotarget-07-54676-s001.pdf]

Circulating exosomes potentiate tumor malignant properties in a mouse model of chronic sleep fragmentation

Supplementary Materials

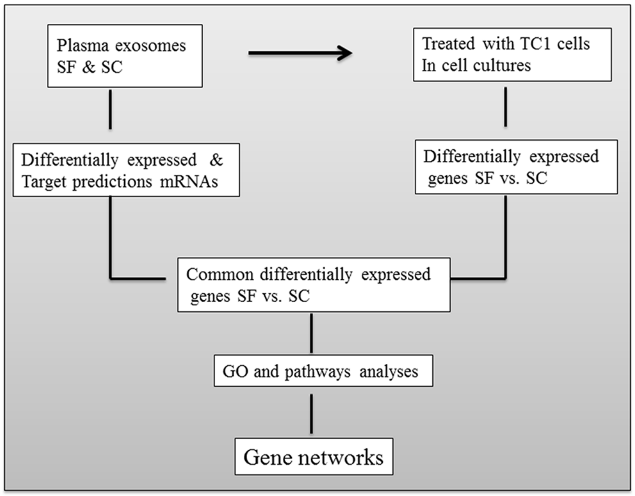

Supplementary Figure S1: Diagram illustrates the integration of miRNA target predictions and differentially expressed mRNAs genes identified in TC1 cells.

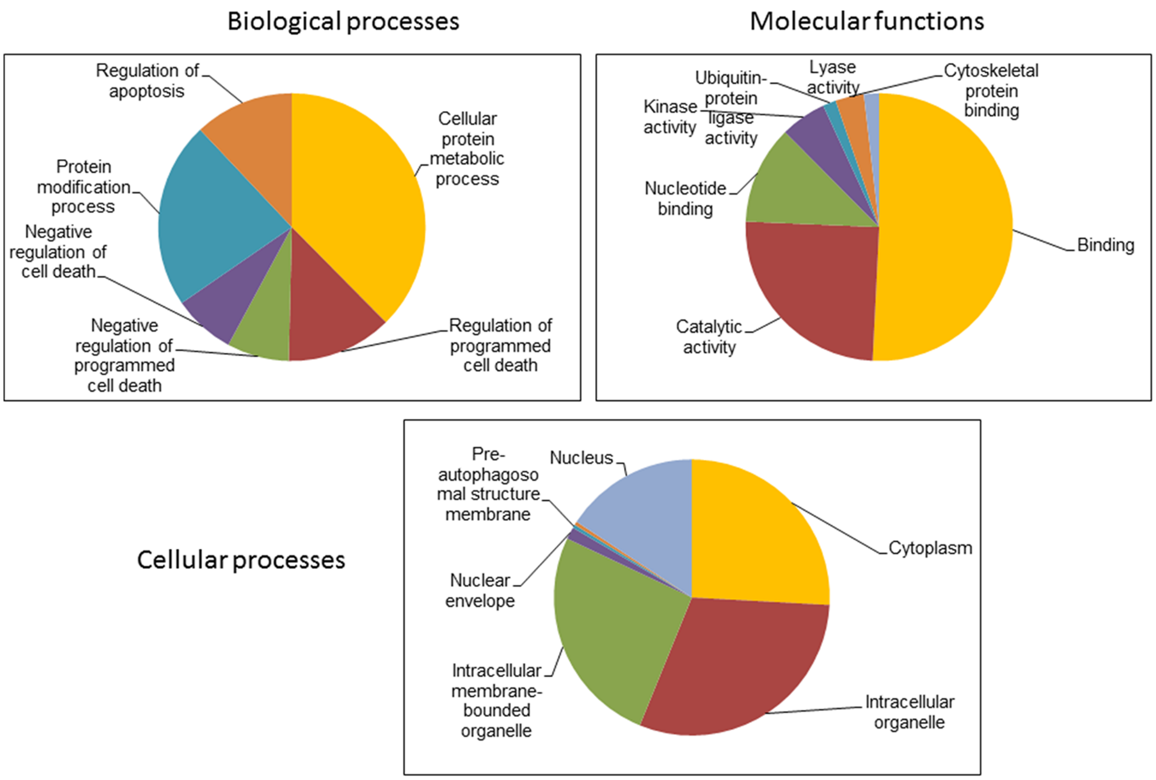

Supplementary Figure S2: Gene ontology of the integrated miRNA target predictions and differentially expressed mRNAs in TC1 cells after treatment with SF or SC-derived plasma exosomes, and include biological processes, molecular functions, and cellular components.

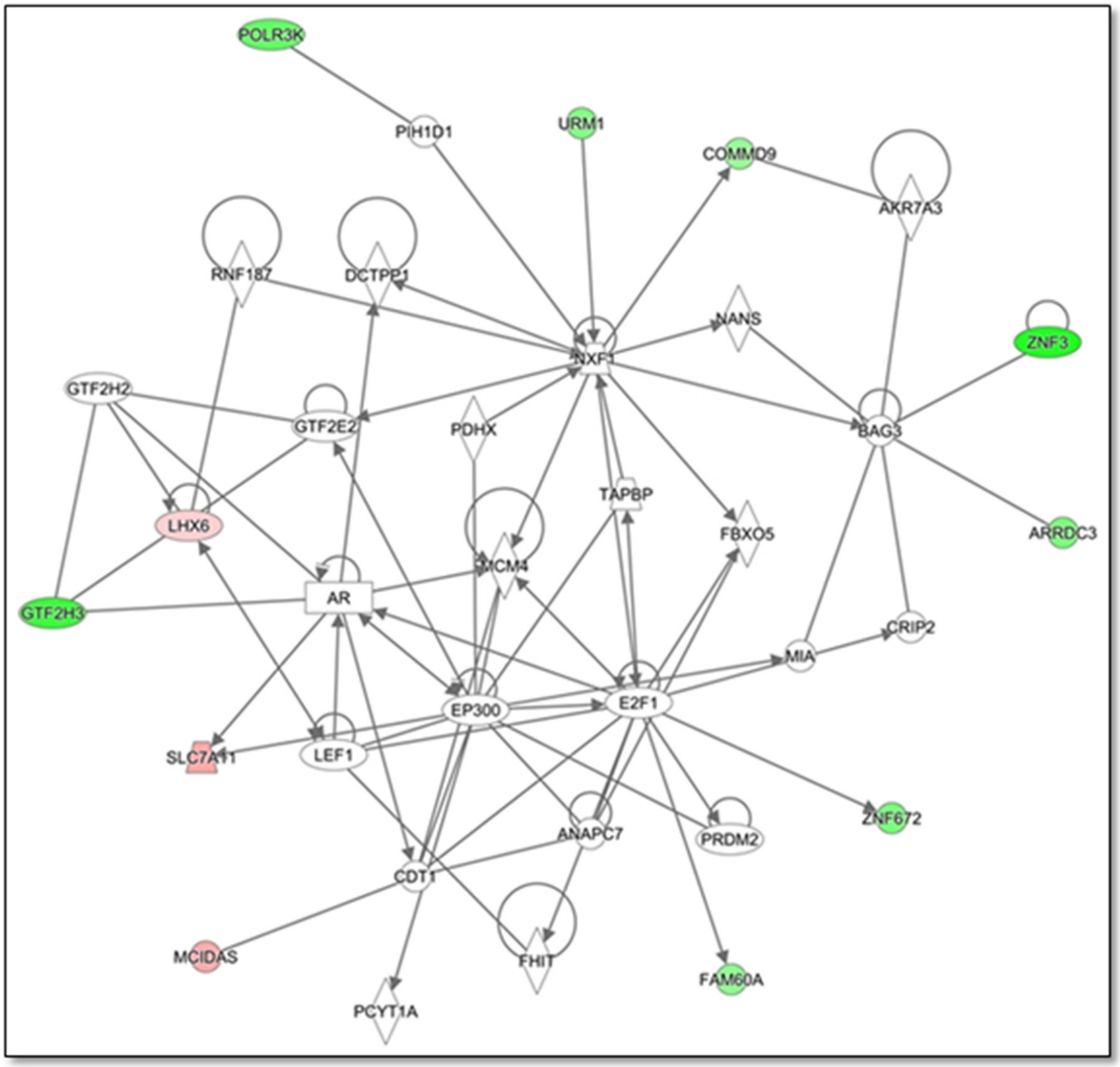

Supplementary Figure S3: Ingenuity pathway analysis illustrating networks derived from cell cycle, reproductive system development and function, and cancer.

Supplementary Table S1: List of the top 50 highly significant differentially expressed mRNA genes identified after TC1 cell treatment with exosomes from SF(−) and SC(−). See Supplementary Table S1

Supplementary Table S2: List of KEGG potential pathways identified in TC1 cells after treatment with SF(−) and SC(−)-derived plasma exosomes

Number	Term	# of genes	Percentage	*p*-value
1	mmu04110:Cell cycle	35	1.36	1.43E–06
2	mmu03040:Spliceosome	30	1.17	1.11E–04
3	mmu04130:SNARE interactions in vesicular transport	13	0.51	7.41E–04
4	mmu00240:Pyrimidine metabolism	23	0.90	9.78E–04
5	mmu00010:Glycolysis/Gluconeogenesis	18	0.70	0.00131123
6	mmu00030:Pentose phosphate pathway	10	0.39	0.00165021
7	mmu05215:Prostate cancer	21	0.82	0.00242472
8	mmu03420:Nucleotide excision repair	13	0.51	0.00245184
9	mmu05214:Glioma	16	0.62	0.00481913
10	mmu04150:mTOR signaling pathway	14	0.55	0.00664147
11	mmu00052:Galactose metabolism	9	0.35	0.00863724
12	mmu05016:Huntington’s disease	33	1.29	0.0089163
13	mmu03410:Base excision repair	11	0.43	0.01255821
14	mmu05218:Melanoma	16	0.62	0.01297079
15	mmu03030:DNA replication	10	0.39	0.01456424
16	mmu00230:Purine metabolism	28	1.09	0.01905853
17	mmu04623:Cytosolic DNA-sensing pathway	13	0.51	0.01951976
18	mmu04120:Ubiquitin mediated proteolysis	25	0.97	0.01957169
19	mmu00190:Oxidative phosphorylation	24	0.94	0.02138265
20	mmu05211:Renal cell carcinoma	15	0.58	0.02528799
21	mmu05222:Small cell lung cancer	17	0.66	0.02983224
22	mmu04730:Long-term depression	15	0.58	0.03159643
23	mmu05210:Colorectal cancer	17	0.66	0.03292027
24	mmu04210:Apoptosis	17	0.66	0.03623648
25	mmu03018:RNA degradation	13	0.51	0.0367422
26	mmu00620:Pyruvate metabolism	10	0.39	0.03892475
27	mmu05200:Pathways in cancer	48	1.87	0.04656503
28	mmu05220:Chronic myeloid leukemia	15	0.58	0.04751684
29	mmu03450:Non-homologous end-joining	5	0.19	0.05023931
30	mmu04010:MAPK signaling pathway	40	1.56	0.05010035

Results show the number of differentially expressed genes (DEGs) in every pathway.

Supplementary Table S3: Differentially expressed plasma exosomal miRNAs in SF(–) and their target genes in TC1 cells

miRNAs	miRNA expression	EntrezGeneID	GeneSymbol	Log fold change	*P* Value	Microarray FDR-p-value
**mmu-miR-5112**	up-regulated	105171	Arrdc3	−0.23	0.0002	0.040
		207921	Fam228b	−0.29	0.0005	0.048
**mmu-miR-5128**	down-regulated	17769	Mthfr	0.24	4E–05	0.023
**mmu-miR-6366**	down-regulated	228026	Pdk1	0.385	0.0002	0.040
		102098	Arhgef18	0.216	0.0002	0.040
		67749	Mgarp	0.734	0.0002	0.041
		214952	Rhot2	0.199	0.0004	0.045
		14235	Foxm1	0.206	0.0005	0.047

Supplementary Table S4: List of transcription factors identified in integrated miRNA gene target predictions and actual differentially expressed genes (DEGs) in TC1 cells treated with exosomes from either SF(−) or SC(−)-derived plasma exosomes

	TF	Ensemble ID	Gene Name
mmu-miR-5128	Cdip1	ENSMUSG00000004071	cell death inducing Trp53 target 1
	Lhx6	ENSMUSG00000026890	LIM homeobox protein 6
	Rreb1	ENSMUSG00000039087	ras responsive element binding protein 1
	Zfp94	ENSMUSG00000074282	zinc finger protein 94
mmu-miR-5112	**TF**	**Ensemble ID**	**Gene Name**
	Arid3a	ENSMUSG00000019564	AT rich interactive domain 3A
	Bhlhe41	ENSMUSG00000030256	basic helix-loop-helix family, member e41
	Csde1	ENSMUSG00000068823	cold shock domain containing E1, RNA binding
	Elf1	ENSMUSG00000036461	E74-like factor 1
	Lef1	ENSMUSG00000027985	lymphoid enhancer binding factor 1
	Maff	ENSMUSG00000042622	v-maf musculoaponeurotic fibrosarcoma oncogene family, protein F
	Rela	ENSMUSG00000024927	v-rel reticuloendotheliosis viral oncogene homolog A
	Rfx3	ENSMUSG00000040929	regulatory factor X, 3
	Tal1	ENSMUSG00000028717	T cell acute lymphocytic leukemia 1
	Tfdp2	ENSMUSG00000032411	transcription factor Dp 2
	Zbtb26	ENSMUSG00000050714	zinc finger and BTB domain containing 26
	Zfp113	ENSMUSG00000037007	zinc finger protein 113
	Zfp266	ENSMUSG00000060510	zinc finger protein 266
	Zfp592	ENSMUSG00000005621	zinc finger protein 592
	Zfp672	ENSMUSG00000049755	zinc finger protein 672
	Zfp777	ENSMUSG00000071477	zinc finger protein 777
	Zik1	ENSMUSG00000030393	zinc finger protein interacting with K protein 1
	Zkscan1	ENSMUSG00000029729	zinc finger with KRAB and SCAN domains 1
mmu-miR-6366	**TF**	**Ensemble ID**	**Gene Name**
	Foxm1	ENSMUSG00000001517	forkhead box M1
	Thap3	ENSMUSG00000039759	THAP domain containing, apoptosis associated protein 3
	Zeb1	ENSMUSG00000024238	zinc finger E-box binding homeobox 1
