# Supplementary material for: Circulating exosomes potentiate tumor malignant properties in a mouse model of chronic sleep fragmentation
Source: Oncotarget. 2016 Jul 13;7(34):54676–90. doi: 10.18632/oncotarget.10578 (PMC5342372; doi:10.18632/oncotarget.10578)
Supplement: Supplementary file 2 [file oncotarget-07-54676-s002.docx]

**Table S1: List of the top 50 highly significant differentially expressed mRNA genes identified after TC1 cell treatment with exosomes from SF(-) and SC(-).**

| **Number** | **EntrezGeneID** | **GeneSymbol** | **P.Value** | **adj.P.Val** | **Gene Name** |
| --- | --- | --- | --- | --- | --- |
| 1 | 654824 | ANKRD37 | 3.2059E-07 | 0.003874592 | ankyrin repeat domain 37 |
| 2 | 30957 | MAPK8IP3 | 1.8712E-06 | 0.006480909 | mitogen-activated protein kinase 8 interacting protein 3 |
| 3 | 17748 | MT1 | 6.0111E-06 | 0.009542171 | metallothionein 1 |
| 4 | 230098 | ARHGEF39 | 6.0192E-06 | 0.009542171 | Rho guanine nucleotide exchange factor (GEF) 39 |
| 5 | 52040 | PPP1R10 | 7.6813E-06 | 0.009542171 | protein phosphatase 1, regulatory subunit 10 |
| 6 | 227648 | SEC16A | 8.1137E-06 | 0.009542171 | SEC16 homolog A, endoplasmic reticulum export factor |
| 7 | 13653 | EGR1 | 8.1455E-06 | 0.009542171 | early growth response 1 |
| 8 | 22040 | TREX1 | 8.6847E-06 | 0.009542171 | three prime repair exonuclease 1 |
| 9 | 17764 | MTF1 | 1.0382E-05 | 0.010456738 | metal response element binding transcription factor 1 |
| 10 | 56463 | SND1 | 1.2982E-05 | 0.012069424 | staphylococcal nuclease and tudor domain containing 1 |
| 11 | 212483 | FAM193B | 3.0634E-05 | 0.023278865 | family with sequence similarity 193, member B |
| 12 | 72345 | AMER1 | 3.4177E-05 | 0.023278865 | APC membrane recruitment 1 |
| 13 | 20304 | CCL5 | 3.4236E-05 | 0.023278865 | chemokine (C-C motif) ligand 5 |
| 14 | 99100 | CEP152 | 3.8204E-05 | 0.023278865 | centrosomal protein 152 |
| 15 | 17769 | MTHFR | 3.9673E-05 | 0.023278865 | 5,10-methylenetetrahydrofolate reductase |
| 16 | 381280 | HJURP | 4.0448E-05 | 0.023278865 | Holliday junction recognition protein |
| 17 | 216345 | ZFC3H1 | 4.6776E-05 | 0.025560974 | zinc finger, C3H1-type containing |
| 18 | 16598 | KLF2 | 4.8643E-05 | 0.025560974 | Kruppel-like factor 2 |
| 19 | 20471 | SIX1 | 5.2977E-05 | 0.025979608 | sine oculis-related homeobox 1 |
| 20 | 13983 | ESR2 | 5.3739E-05 | 0.025979608 | estrogen receptor 2 (beta) |
| 21 | 223864 | RAPGEF3 | 6.0976E-05 | 0.028016729 | Rap guanine nucleotide exchange factor (GEF) 3 |
| 22 | 66116 | CML1 | 6.9217E-05 | 0.028556867 | camello-like 1 |
| 23 | 433182 | ENO1B | 7.2407E-05 | 0.028556867 | enolase 1B, retrotransposed |
| 24 | 228359 | ARHGAP1 | 7.3931E-05 | 0.028556867 | Rho GTPase activating protein 1 |
| 25 | 14087 | FANCA | 7.7899E-05 | 0.028556867 | Fanconi anemia, complementation group A |
| 26 | 217082 | HLF | 8.0335E-05 | 0.028556867 | hepatic leukemia factor |
| 27 | 383592 | GM1305 | 8.4125E-05 | 0.029049414 | predicted gene 1305 |
| 28 | 16409 | ITGAM | 8.9571E-05 | 0.029349164 | integrin alpha M |
| 29 | 104263 | KDM3A | 9.143E-05 | 0.029349164 | lysine (K)-specific demethylase 3A |
| 30 | 80879 | SLC16A3 | 9.5614E-05 | 0.029630434 | solute carrier family 16 (monocarboxylic acid transporters), member 3 |
| 31 | 74204 | XPO6 | 0.00010674 | 0.031640465 | exportin 6 |
| 32 | 74747 | DDIT4 | 0.00010734 | 0.031640465 | DNA-damage-inducible transcript 4 |
| 33 | 27981 | RSRP1 | 0.00012653 | 0.036409604 | arginine/serine rich protein 1 |
| 34 | 54393 | GABBR1 | 0.00013602 | 0.038232356 | gamma-aminobutyric acid (GABA) B receptor, 1 |
| 35 | 105171 | ARRDC3 | 0.00015746 | 0.040378389 | arrestin domain containing 3 |
| 36 | 240038 | GM4944 | 0.00016066 | 0.040378389 | predicted gene 4944 |
| 37 | 100041230 | HIST1H4M | 0.00016801 | 0.040378389 | histone cluster 1, H4m |
| 38 | 228026 | PDK1 | 0.00017155 | 0.040378389 | pyruvate dehydrogenase kinase, isoenzyme 1 |
| 39 | 102098 | ARHGEF18 | 0.00017194 | 0.040378389 | rho/rac guanine nucleotide exchange factor (GEF) 18 |
| 40 | 380608 | TAGAP1 | 0.00017271 | 0.040378389 | T cell activation GTPase activating protein 1 |
| 41 | 19044 | PPOX | 0.0001741 | 0.040378389 | protoporphyrinogen oxidase |
| 42 | 50878 | STAG3 | 0.00017436 | 0.040378389 | stromal antigen 3 |
| 43 | 268481 | KRT222 | 0.00017635 | 0.040378389 | keratin 222 |
| 44 | 240084 | CCHCR1 | 0.00018374 | 0.040378389 | coiled-coil alpha-helical rod protein 1 |
| 45 | 217057 | PTRH2 | 0.00018515 | 0.040378389 | peptidyl-tRNA hydrolase 2 |
| 46 | 102414 | CLK3 | 0.00018623 | 0.040378389 | CDC-like kinase 3 |
| 47 | 432628 | MFSD2B | 0.00019788 | 0.040378389 | major facilitator superfamily domain containing 2B |
| 48 | 16007 | CYR61 | 0.00019826 | 0.040378389 | cysteine rich protein 61 |
| 49 | 353310 | ZFP703 | 0.00019931 | 0.040378389 | zinc finger protein 703 |
| 50 | 108077 | SKIV2L | 0.00020362 | 0.040378389 | superkiller viralicidic activity 2-like |
